# The Ancient World; or Picturesque Sketches of Creation

**Published:** 1847-07

**Authors:** 


ART. III.—*The Ancient World; or Picturesque Sketches of Creation.* By D. T. ANSTED, M.A., F.R.S., F.G.S.; Professor of Geology in King's College, London, &c. &c.—*London*, 1847. Small 8vo, pp. 408, with numerous Wood Engravings.

WE know few instances in which a book has been more completely adapted to supply an existing want, than the one now before us. Every educated man in the present day is expected to know something of the past history of the globe on which we live; but the difficulty of obtaining a general and philosophical view of that history from any treatises previously extant, without having the attention distracted by a mass of details which are valuable only to those who are systematically pursuing the subject, must be well known to all who have made the attempt. Now the object of this treatise of Professor Ansted's is to communicate in a simple form to the general reader the chief *results* of geological investigation; more especially such as have reference to the living inhabitants of the earth at different epochs. A succinct outline is given of those great physical changes, which have produced that diversity of conditions to which we owe the successive extinction of certain races and the opportunity for the introduction of others; but the chief attention is bestowed upon the probable characters and habits of the denizens of the globe at different periods, as deduced from the study of those fossil remains which have been preserved to our own time. The most interesting of these are described in such a manner as to bring them before the mind's eye, with much of the vividness of conception which we derive from the accounts of some new region explored by an adventurous traveller, who has personally witnessed what he depicts. And although it may seem as if it were necessary to draw somewhat upon the imagination in such delineations, yet we believe that Professor Ansted has in no instance gone beyond the most legitimate deductions from the wonderfully complete evidence, which a careful examination of the records of the ancient world brings under the review of the Geologist. As he justly remarks, there are not wanting other instances in which conclusions perfectly satisfactory have been arrived at, and histories prepared, without the existence of any written documents whatever. The domestic manners of the ancient Egyptians are now known to us fully as well as those of their existing representatives, through the medium of those pictorial delineations of themselves which they have left us in such abundance; and from similar delineation, we are learning more and more of another ancient people, the Etrurians, whose very existence could only be inferred with hesitation from direct historical testimony, but of whose high civilization and proficiency in the arts of life, at a period long anterior to that usually assigned to the foundation of Rome, new evidence is continually being acquired, especially from sepulchral monuments. And if we look with interest to the discoveries which are being made from time to time, with reference to the past history of fragmentary portions of our own race, we surely need not entertain a less degree of curiosity with regard to those gigantic changes of which our globe has been so often the subject, previously to man's introduction upon its surface; whilst the novelty of the forms of animated beings which have been its successive tenants, their evident adaptation to conditions of existence very remote from those which now prevail, and the abundant materials for speculation as to the structure of those parts which are lost to us, present one of the most attractive fields that can be conceived for the wholesome and pleasurable exercise of the mind.

We cannot too strongly recommend this volume to our readers; its exterior is elegant, and its interior attractive to a superficial glance; and we are confident that its contents, when carefully studied, will not disappoint the first impression.

